# Molecular and Biochemical Differences of the Tandem and Cold-Adapted PET Hydrolases Ple628 and Ple629, Isolated From a Marine Microbial Consortium

**DOI:** 10.3389/fbioe.2022.930140

**Published:** 2022-07-21

**Authors:** Ingrid E. Meyer Cifuentes, Pan Wu, Yipei Zhao, Weidong Liu, Meina Neumann-Schaal, Lara Pfaff, Justyna Barys, Zhishuai Li, Jian Gao, Xu Han, Uwe T. Bornscheuer, Ren Wei, Başak Öztürk

**Affiliations:** ^1^ Junior Research Group Microbial Biotechnology, Leibniz Institute DSMZ—German Collection of Microorganisms and Cell Cultures, Braunschweig, Germany; ^2^ Tianjin Institute of Industrial Biotechnology, Chinese Academy of Sciences, Tianjin, China; ^3^ College of Biotechnology, Tianjin University of Science and Technology, Tianjin, China; ^4^ Research Group Metabolomics, Leibniz Institute DSMZ—German Collection of Microorganisms and Cell Cultures, Braunschweig, Germany; ^5^ Junior Research Group Plastic Biodegradation, Institute of Biochemistry, Department of Biotechnology and Enzyme Catalysis, University of Greifswald, Greifswald, Germany; ^6^ Department of Biotechnology and Enzyme Catalysis, Institute of Biochemistry, University of Greifswald, Greifswald, Germany

**Keywords:** biodegradable plastics, PETase-like enzymes, tandem PETases, marine biodegradation, PETase activity

## Abstract

Polybutylene adipate terephthalate (PBAT) is a biodegradable alternative to polyethylene and can be broadly used in various applications. These polymers can be degraded by hydrolases of terrestrial and aquatic origin. In a previous study, we identified tandem PETase-like hydrolases (Ples) from the marine microbial consortium I1 that were highly expressed when a PBAT blend was supplied as the only carbon source. In this study, the tandem Ples, Ple628 and Ple629, were recombinantly expressed and characterized. Both enzymes are mesophilic and active on a wide range of oligomers. The activities of the Ples differed greatly when model substrates, PBAT-modified polymers or PET nanoparticles were supplied. Ple629 was always more active than Ple628. Crystal structures of Ple628 and Ple629 revealed a structural similarity to other PETases and can be classified as member of the PETases IIa subclass, α/β hydrolase superfamily. Our results show that the predicted functions of Ple628 and Ple629 agree with the bioinformatic predictions, and these enzymes play a significant role in the plastic degradation by the consortium.

## 1 Introduction

Biodegradable plastics have been introduced to the market as an ecologically-friendly alternative to recalcitrant plastics (Platt, 2006). These are especially used in applications where the desired product is intended to have a short life (e.g., single-use plastics), and recycling is not feasible or possible (Wei et al., 2020). Difficulties in recycling might appear when plastics contain other types of materials (e.g., paper and other organic matter) (Hopewell et al., 2009; Hahladakis and Iacovidou, 2019). One such biodegradable polymer is polybutylene adipate co-terephthalate (PBAT). PBAT is an aliphatic-aromatic co-polyester synthesized by polycondensation of terephthalic acid (T), adipic acid (A) and 1, 4-butanediol (B) (Jian et al., 2020). Due to its composability and similarity in mechanical properties, it is marketed as a biodegradable alternative to polyethylene and is widely used to manufacture agricultural mulch films, plastic bags, and paper coatings (BASF, 2022). At the same time, PBAT can be used as a carbon source due to its bioavailability and its low crystallinity makes it accessible to enzymatic degradation (Tiso et al., 2022). The formed products T, A, and B can be further up-cycled to produce new materials (Tiso et al., 2022).

There are numerous known enzymes with PBAT hydrolytic activity. These are cutinase-like serine hydrolases (Kleeberg et al., 2005; Thumarat et al., 2012; Perz et al., 2016b) that originate from the terrestrial environment or the aquatic environment (Wallace et al., 2017). Poly (ethylene terephthalate) (PET) is an aromatic polyester, and PBAT is an aliphatic-aromatic polymer with a terephthalate-diol component. They both share some structural similarities in relation to the T-diol component. PETases can degrade PET (Yoshida et al., 2016) and are assumed to degrade PBAT polymers as well (Kawai et al., 2020). Some PBAT-degrading enzymes can also degrade PET, albeit with less efficiency (Gouda et al., 2002; Kleeberg et al., 2005), possibly due to the high proportion of aromatic moieties in PET (Marten et al., 2005) and higher crystallinity (Wei et al., 2022). Enzymatic PBAT degradation yields a mixture of the terephthalate-butanediol monoester (BT) intermediate and monomers (Perz et al., 2016b). Similarly, the degradation of PET yields a mixture of mono-(2-hydroxyethyl) terephthalic acid (MHET), bis (2-hydroxyethyl) terephthalate (BHET) and monomers (Yoshida et al., 2016). Not all PBAT-degrading enzymes can degrade the BT intermediate: Thc_Cut1 cutinase from *Thermobifida cellulosilytica* can degrade PBAT efficiently to its monomers (Perz et al., 2016b), while PpEst from *Pseudomonas alcaligenes* cannot degrade the BT intermediate and is inhibited by it (Wallace et al., 2017). Although HiC, a cutinase from *Humicola insolens*, is not inhibited by BHET and MHET when incubated with PET (Eugenio et al., 2021), the release of BT after degradation of a PBAT-blend by HiC seems to inhibit the activity of the enzyme (Perz et al., 2016b). Among these enzymes, only PpEst (Wallace et al., 2017) originates from the aquatic environment. PE-H from the marine bacterium *Pseudomonas aestusnigri* was shown to degrade PET, but the activity of this enzyme was not tested on PBAT (Bollinger et al., 2020).

We have previously identified two putative PETase-like enzymes (Ples) from a marine microbial consortium, named “I1,” which grows on a commercial PBAT-blend film (ecovio® FT 2341) as the only carbon source (Meyer-Cifuentes et al., 2020). These enzymes were named Ple628 and Ple629. The genes encoding for these enzymes are in tandem, with a few hundred bases and no open reading frames in between. The enzymes were characterized as α/β hydrolases, and had the lipase/esterase signature GXSXG (Ollis et al., 1992; Arpigny and Jaeger, 1999). They are closely related to each other (74% amino acid (aa) identity) and to know PETases such as the *Ideonella sakaiensis* PETase (*Is*PETase) (A0A0K8P6T7, GAP38373) (Yoshida et al., 2016), leaf compost cutinase LCC (AEV21261.1) (Sulaiman et al., 2012) and *Thermobifida fusca* cutinase Thf42_Cut1 (ADV92528.1) (Herrero Acero et al., 2011) (45–50% aa identity). Metatranscriptomic and proteomic analyses showed that the genes encoding these enzymes were highly upregulated during incubation with the commercial PBAT-blend, and the production of the proteins increased, especially in the biofilm growing on the plastic film (Meyer-Cifuentes et al., 2020). We postulated that these enzymes perform the depolymerization of ecovio^®^ FT 2341, and in the process release BT (named previously as “Bte”) as well as aliphatic oligomers (Meyer-Cifuentes et al., 2020). The BT then gets degraded to B and T by a third enzyme, Mle046, whose function we have recently verified after recombinant expression and biochemical analysis (Meyer-Cifuentes and Öztürk, 2021). The functions of Ple628 and Ple629, their differences and biochemical properties, however, remained unverified.

In this study, we recombinantly expressed, purified and characterized Ple628 and Ple629 as marine PET hydrolases. In this framework, we comparatively analyze their kinetic properties, degradation products, and activity on smaller PBAT oligomers (supplied as powder), ecovio®FT, PBAT, and PBSeT (supplied as film), as well as PET nanoparticles. We also elucidate and analyze the structures of both proteins by crystallography.

## 2 Results

### 2.1 Ple628 and Ple629 Purification and Identification

**FIGURE 1:**
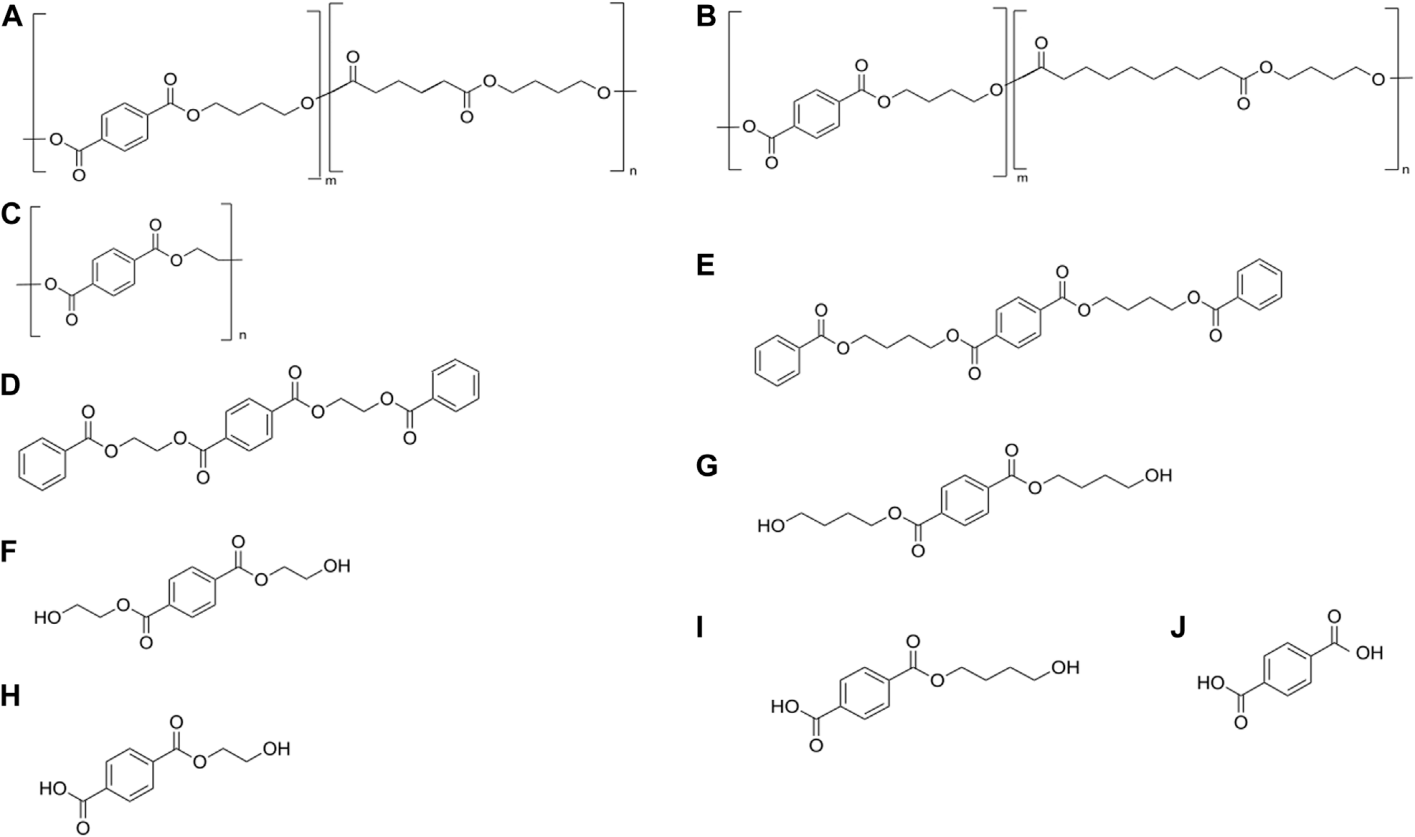
Substrates and degradation products of Ples. **(A)** PBAT, **(B)** PBSeT, **(C)** PET, **(D)** 3-PET, **(E)** 3-PBT, **(F)** BHET, **(G)** BHBT, **(H)** MHET, **(I)** BT, **(J)** T. ecovio® FT is a blend of **(A,B)**, with a small amount of added polylactic acid (less than 10%).

The Ple628 and Ple629 sequences were retrieved from a previous study (Meyer-Cifuentes et al., 2020). Phylogenetic analysis of Ple628 and Ple629 and other PETase-like enzymes were described in more detail in the previously mentioned study (Meyer-Cifuentes et al., 2020). These two enzymes were phylogenetically classified as type IIa PETases (Supplementary Figure S1). Additionally, the *ple628* and *ple629* sequences used in this study were codon optimized for recombinant protein expression. We successfully produced and purified Ple628 and Ple629 from *E. coli* Origami (DE3). The sizes were similar to those predicted bioinformatically (32.6 and 31.7 kDa for Ple628 and Ple629, respectively) (Supplementary Figure S2). The purest fractions of each protein were pooled and used in this study. The substrates and degradation products that were tested and measured in this study are given in Figure 1.

### 2.2 Kinetic Parameters of Ple628 and Ple629

To further analyze the differences observed between Ple628 and Ple629, we determined their enzymatic kinetic parameters, *K*m and *k*cat. These parameters were obtained by measuring the hydrolysis of (4-nitrophenyl) acetate (*p*NPA). We observed that Ple628 had a higher affinity for the substrate *p*NPA compared to Ple629. Ple628 required 1.8 mM of the substrate to saturate half of the Ple628 present in the reaction, while Ple629 required 2.1 mM. However, the calculated turnover rate was higher for Ple629 (284.5 min^−1^) than for Ple628 (269.9 min^−1^). This observation indicates that Ple629 hydrolyses the small substrate pNPA somewhat faster than Ple628.

The kinetic parameters of Ple629 on ecovio^®^FT film were calculated by plotting different Ple629 concentrations against the rates of ecovio^®^FT degradation [inverse Michaelis-Menten (^inv^MM)]. This method allows the analysis of enzymatic activities and subsequent calculation of kinetic parameters of enzymes acting on insoluble substrates (Bååth et al., 2021; Vogel et al., 2021). For the analysis, we tested concentrations from 0.5 to 6.5 µM of Ple629 and measured the formation of BT and T. The highest degradation rate was achieved with 4.5 µM of Ple629. The ^inv^ *K*m is 1.86 µM and the ^inv^ *k*cat is 0.017 h^−1^.

### 2.3 Activity of Ple629 and Ple628 on Small Terephthalate-Esters

We analyzed the range of action of both enzymes, Ple629 and Ple628, on oligomeric model substrates of PBAT and PET. The substrates tested were bis(2-(benzoyloxy)ethyl) terephthalate (3-PET), bis(4-(benzoyloxy)butyl) terephthalate (3-PBT), bis(hydroxyethyl) terephthalate (BHET) and bis(4-hydroxybutyl) terephthalate (BHBT). All these model substrates were supplied as powder. The formation of the degradation products, BT, MHET, and T, was monitored after 1, 2, and 24 h of incubation. For the degradation of BHET, an additional sampling point after 30 min was included.

**FIGURE 2:**
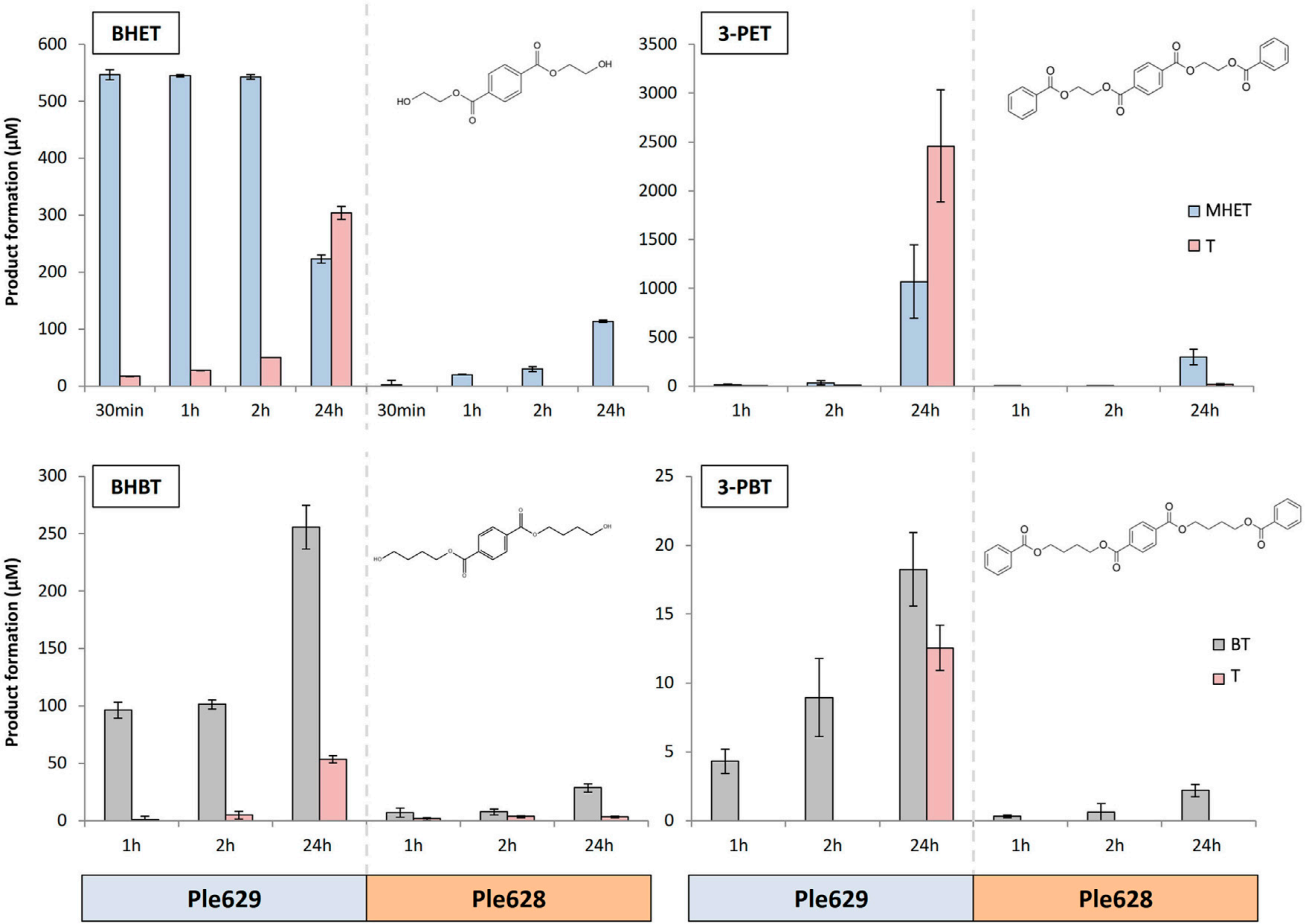
Degradation of small terephthalate-esters by Ples. Formation of T (dark pink) and MHET (light blue) or BT (light gray) (in µM) after degradation of BHET, BHBT, 3-PET, and 3-PBT by Ple628 and Ple629. The degradation of BHET and 3-PET leads to the formation of MHET and T and the degradation of BHBT and 3-PBT to the formation of BT and T. The molecular structure of each substrate is shown at the uppermost right corner of each bar plot. The sampling points are shown at 30 min, 1 h, 2h, and 24 h. The error bars indicate the standard deviation (*n* = 3).

We observed that Ple629 could degrade 3-PET and, to some extent, 3-PBT. Ple629 incubation with 1 mg of 3-PBT released a maximum amount of 35.9 ± 4.1 µM of BT after 24 h (Figure 2). In comparison, when 1 mg of 3-PET was used as the substrate, more than 2,000 µM of MHET was formed. The catalysis of 3-PET by Ple629 also yielded high amounts of T after 24 h of incubation (approximately 1,900 µM of T). Formation of T after 3-PBT degradation by Ple629 was only detected after 24 h of incubation. Conversely, Ple629 incubation with 3-PET yielded T readily after 1 h of incubation. Ple628 could also degrade 3-PET but to a lesser extent than Ple629. Relative to MHET formation after 3-PET degradation by Ple629, we detected less than 30% of MHET formation (601.6 ± 374.1 µM of MHET) when Ple628 was incubated with 3-PET. Consequently, Ple628 produced low amounts of T (13.4 ± 8.0 µM) which were only detected after 24 h of incubation. Similar to Ple629, Ple628 had poor activity on 3-PBT. With Ple628, a maximum of 4.7 ± 0.8 µM of BT was released after 24 h of incubation. Only 0.6 ± 0.5 µM of T was detected when Ple628 was incubated after 24 h with 3-PBT.

The intermediates of 3-PET and 3-PBT degradation, BHET and BHBT, respectively, were also degraded by Ple628 and Ple629. Ple629 achieved maximum MHET formation after 2 h of incubation with BHET (758.2 ± 60.9 µM) (Figure 2). T was also formed after 30 min, and its production increased throughout the incubation. The maximum amount of T produced was 194.7 ± 4.3 µM after 24 h. Compared to BHET degradation, Ple629 showed lower activity with BHBT. With BHBT, the maximum amounts of BT and T formed after 24 h of incubation were 513.2 ± 31.4 and 42.3 ± 1.9 µM, respectively. Conversely, the incubation of Ple628 with the substrate BHET produced less MHET than with Ple629. After 30 min and 24 h, only 8.4 and 112.9 ± 2.0 µM of MHET were formed, respectively. We did not detect T production at any time point, which indicates that MHET was not further hydrolyzed to T and ethylene glycol. Compared to MHET formation after BHET degradation by Ple628, low amounts of BT were formed when BHBT was used as the substrate. After 24 h of incubation, Ple628 could form only 56.93 ± 5.9 µM of BT. Traces of T were detected at each time point (1.6 ± 1.0 to 2.1 ± 0.5 µM T).

### 2.4 Temperature Optima of Ple628 and Ple629

**FIGURE 3:**
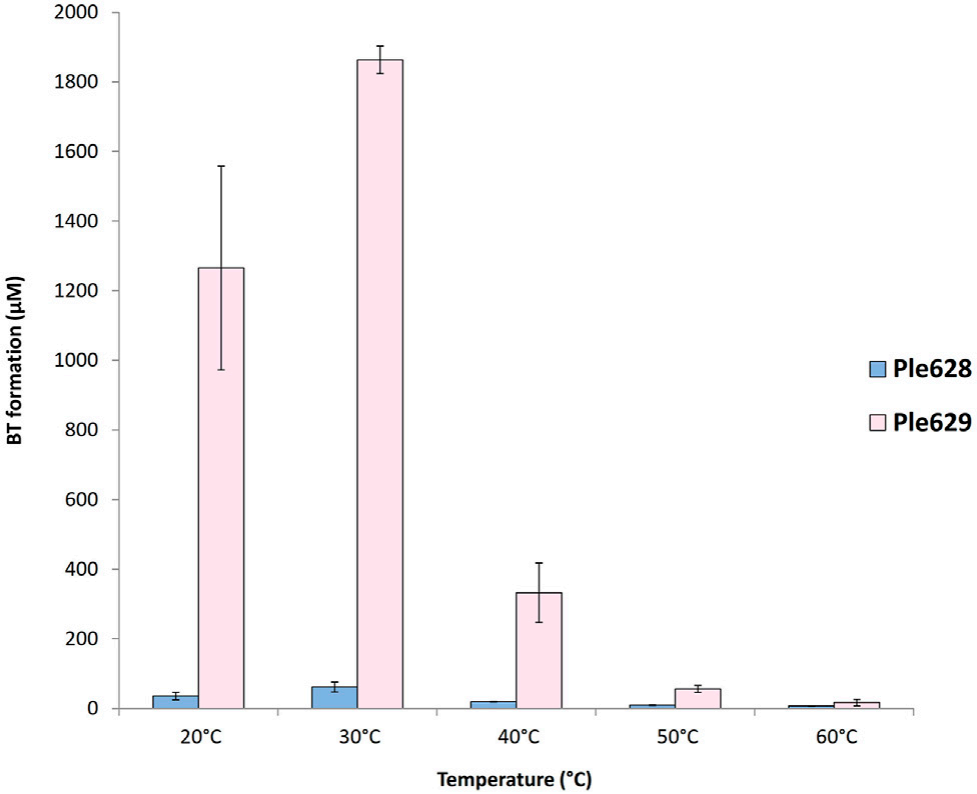
Ples temperature optimum of incubation. Formation of BT (in µM) after degradation of ecovio^®^FT by Ple628 and Ple629. Ple628 and Ple629 are shown in blue and pink, respectively. The tested temperatures were 20, 30, 40, 50, and 60°C. The error bars indicate the standard deviation (*n* = 3).

Ple628 and Ple629 were incubated at different temperatures for 72 h to find the temperature range that gave the highest activity. When using ecovio^®^FT as film, we found that Ple629 and Ple628 are mostly active at 30°C (Figure 3). Relative to the Ples enzymatic activity observed at 30°C, the Ples retained most of their activity when the incubation temperature was reduced to 20°C. At 20°C, the Ple629 and Ple628 produced (relative to 30°C) 68 and 58% of BT, respectively. On the contrary, temperatures above 30°C negatively affected the activity of both enzymes. When Ple628 or Ple629 were incubated at 40°C, the BT formation dropped to 31% and 18% compared to the product formation at 30°C, respectively. At 60°C, the highest temperature tested, the BT formation dropped further to 12 and 0.9% for Ple628 and Ple629, respectively. T was also formed, but only in the presence of Ple629. Specifically, T was detected when Ple629 was incubated at 20, 30 and 40°C exclusively. At higher temperatures, T was undetectable. No products were released in the absence of an enzyme at temperatures ≥ of 50°C. This indicates that the substrate ecovio^®^FT and the intermediate BT are not affected by auto-hydrolysis at 50°C.

The melting temperatures (Tms) of Ple628 and Ple629 were determined to be 41.4°C and 38.1°C in PBS by nanoDSF, and 47.1°C and 43.2°C by DSC (Supplementary Table S1), respectively.

### 2.5 Activity of Ple629 and Ple628 on Biodegradable Plastic Films and PET Nanoparticles

**FIGURE 4:**
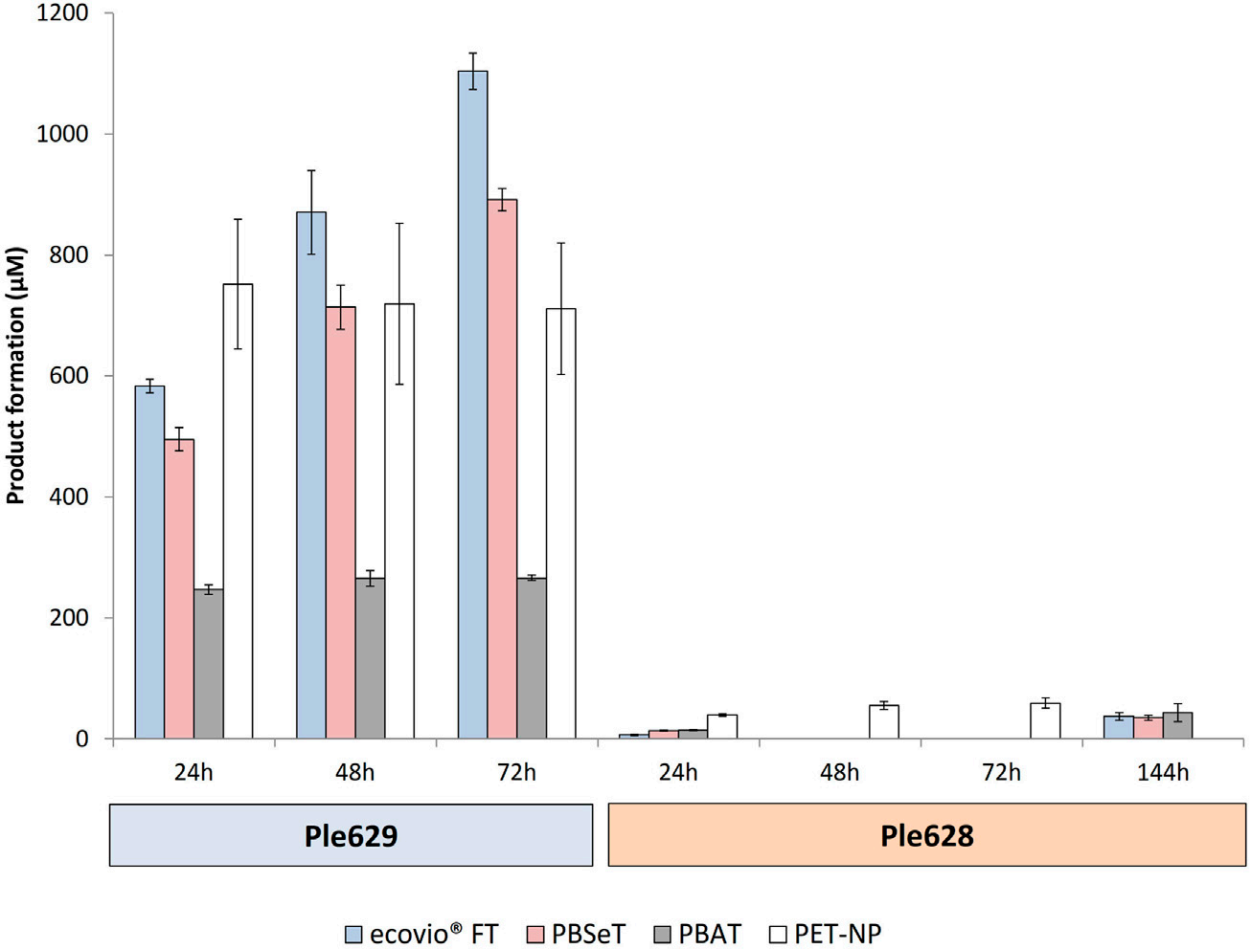
Degradation of biodegradable plastics and PET-NP by Ples. Formation of BT or MHET (in µM) after degradation of ecovio^®^FT (light blue), PBAT (light gray), PBSeT (dark pink) and PET-NP (white) by Ple629 and Ple628. The degradation of biodegradable plastics and PET-NP leads to the formation of BT and MHET, respectively. The sampling points are shown at 24, 48, 72, and 144 h. The error bars indicate the standard deviation (*n* = 3).

The activity of the enzymes Ple629 and Ple628 was tested on PBAT, PBSeT, ecovio^®^FT films and on PET nanoparticles (PET-NP) at 30°C over time. We observed that both enzymes, Ple628 and Ple629, can hydrolyze all tested polymers. Compared to Ple629, however, Ple628 was less active on all tested polymer substrates. Ple628 produced low amounts of BT, the intermediate product of ecovio^®^FT degradation, in the presence of PBAT, PBSeT or ecovio^®^FT. Even after 144 h of incubation, Ple628 produced less than 50 µM of BT with either of the tested polymers. Ple629 yielded 1,103.6 ± 29.8, 891.5 ± 18.6 and 265.9 ± 4.2 µM of BT after 72 h with ecovio^®^FT, PBSeT and PBAT (Figure 4). Comparable to the assays with the biodegradable plastics, Ple628 produced low amounts of MHET after PET-NP degradation. After 72 h, Ple628 released only 52.9 ± 1.1 µM of MHET, compared to 785.9 ± 27.8 µM of MHET released by Ple629 (Figure 4). The product formation did not significantly increase between 48 and 72 h of incubation for any of the enzymes.

To some extent, Ple629 could degrade the biodegradable plastic films to T. After 72 h, 35.1 ± 2.5, 31.2 ± 1.6 and 18.9 ± 1.3 µM of T were detected after degradation of ecovio^®^FT, PBSeT, and PBAT, respectively (Supplementary Figure S3). Surprisingly, Ple629 released high amounts of T after PET-NP degradation, reaching a maximum of 736.3 ± 12.0 µM after 72 h of incubation. As expected, lower amounts (9.5 ± 1.3 µM) of T were released in the presence of Ple628. When the plastic polymers were supplied as the substrate, less than 1 µM of T was formed.

Interestingly, with PBAT as the substrate, the production of BT by Ple629 ceased after 24 h and remained the same between 48 and 72 h (265 ± 0.03 µM). This observation might indicate that Ple629 has limited activity on PBAT compared to the other polymers tested (Figure 4). By comparing the amount of products (BT and T) released in the presence of Ple629, we could also observe that the enzyme degrades more efficiently ecovio^®^FT over PBSeT and PBAT. We detected insignificant degradation of untreated (without addition of Ple629 or Ple628) ecovio^®^FT and PBSeT to BT (3.5–11.0 µM BT released after 72 h).

We additionally semi-quantitatively analyzed the release of T and other products by LC-MS after degradation of PBAT and PBSeT by Ple629 or Ple628. A, T and sebacic acid (S), the building blocks of the tested PBAT-like films and mixtures of these monomers were detected (Supplementary Figure S4). The amount of each monomer increased in each reaction after 72 h of incubation. When Ple628 was incubated with either PBAT or PBSeT, T was detected only after 72 h. Conversely, T was readily produced and detected after 24 h when PBAT or PBSeT were incubated with Ple629. The amount of T produced after 72 h was 75-times higher after degradation by Ple629 than Ple628 for both plastics. BT was the only compound detected consistently in all assays. The amount of BT increased 3 times between 24 and 72 h of degradation when PBAT or PBSeT was incubated with Ple629. Approximately 1.5 times more BT was detected when PBSeT was used as the substrate than when PBAT was used. For Ple628, no significant change in BT concentration was observed between these time points. The total amount of BT produced by Ple628 was 6 times less than Ple629 when PBAT was used as a substrate, and 10 times less when PBSeT was used. A mixture of adipate-butanediol (A + B) was only detected after degradation of PBAT with Ple628. Sebacic acid + butanediol (S + B) was not detected in any assay. S and A were both detected in experiments supplied with PBAT and PBSeT, even though PBAT does not contain S and PBSeT does not contain A. This is likely due to cross contamination during the successive extrusion of the films for the study.

### 2.6 Inhibition of ecovio^®^FT Degradation by BT

**FIGURE 5:**
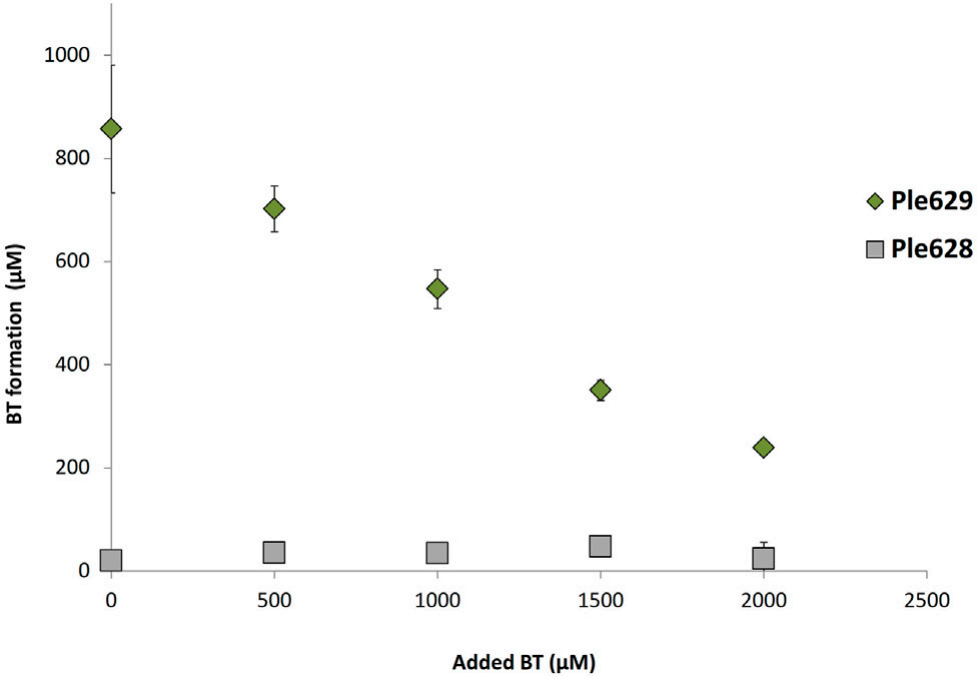
Inhibition of ecovio^®^FT degradation by BT. Analysis of ecovio^®^FT degradation by Ple629 and Ple628 to BT (in µM) at different inhibitory concentrations of BT. Ple629 is shown as dark green diamonds and Ple628 as gray squares. The tested BT concentrations were 0 (control), 500; 1,500; and 2,000 µM. The error bars indicate the standard deviation (*n* = 3).

Films of ecovio^®^FT were incubated with Ple628 and Ple629 and with increasing concentrations of BT. Previously, homologous enzymes with PET hydrolyzing activities were found to be inhibited by the intermediates BHET and MHET (Barth et al., 2015, 2016). Therefore, we aimed to determine if the activities of Ple628 and Ple629 are also inhibited by intermediates of ecovio^®^FT degradation, specifically BT. Without the addition of BT, the degradation of 5 mg of ecovio^®^FT yielded 204 mg/L of BT. Reactions supplied with more than 1,000 µM of additional BT released less than 50% (Figure 5) of the BT formed by subtracting the amount of supplementary BT. This observation indicates that Ple629 is inhibited by this degradation product. As shown before, Ple628 already had very low activity on ecovio^®^FT and released only small amounts of BT with or without the addition of external BT. Because of this, we could not assess product inhibition of Ple268 by BT.

### 2.7 Ple628 and Ple629 Sequences and Crystal Structures Reveal Similarity to Known PETases

**FIGURE 6:**
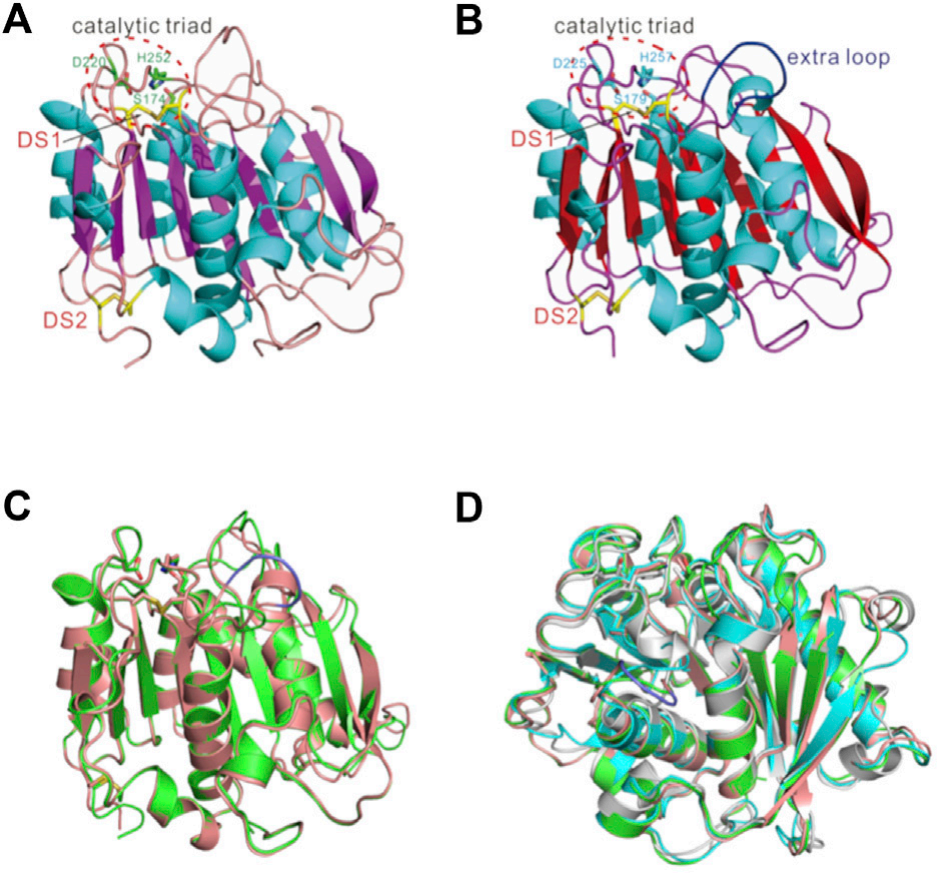
Crystal structures of **(A)** Ple628, **(B)** Ple629, superimposed structures of **(C)** Ple628 (green) and Ple629 (salmon), and **(D)** Ple628, Ple629, *Is*PETase (cyan), and PE-H (gray). The catalytic triads, Ple629 extended loop (blue), and disulphide bonds (labeled DS, yellow) are depicted.

For structural determination of Ple628 and Ple629, the signal peptide sequences (M1-A25 for Ple628, and M1-A27 for Ple629) were removed for the production of the core domain of the protein. As predicted from the sequence homology to the lipase and cutinase families, Ple628 and Ple629 both belong to the α/β hydrolase superfamily, and the central twisted β-sheet is formed by nine mixed β-strands (β1–β9) and surrounded by eight α-helices (Figures 6A,B). The catalytic triad S174/S179-D220/D225-H252/H257 was conserved among all PETase-like proteins (positions in Ple628/Ple629, respectively). Like previously described for *Is*PETase and PE-H (Joo et al., 2018; Bollinger et al., 2020; Wei et al., 2022), Ple628 and Ple629 form two disulfide bridges, DS1(C217-C254/C222-C259) and DS2(C288-C305/C297-C314) (positions in Ple628/Ple629, respectively). The data collection and refinement statistics are given in Supplementary Table S2.

The main structural difference between Ple628 and Ple629 was the presence of an extended loop in Ple629, which is longer than the one present in Ple628, with the four extra residues S264, G265, F266, and G267 (Figure 6C). Ple628 has a shorter extended loop, similar to the *Is*PETase (Joo et al., 2018) and PE-H (Bollinger et al., 2020) (Figure 6D). The root-mean-square deviation (RMSD) of atomic positions of Ple628, Ple629, and previously characterized PETases and other hydrolases confirmed that Ple629 is more similar to mesophilic PETases such as PE-H and *Is*PETase than it is to LC-cutinase and Est119 (Supplementary Table S3). Interestingly, Ple629 is structurally more similar to the marine PETase PE-H than to Ple628.

## 3 Discussion

In this study, we purified and biochemically characterized two tandem PETases from a marine microbial consortium that grows on the aliphatic-aromatic co-polyester ecovio^®^FT. These PETases degrade a broad range of aromatic and aromatic-aliphatic polyesters at moderate temperatures and perform the first step of ecovio^®^FT degradation by the marine consortium that they originate from (Meyer-Cifuentes et al., 2020).

To understand the mode of action and differences between Ple628 and Ple629, derivatives of PBAT and smaller oligomeric model substrates were used. The other PETases and PBAT hydrolases that are referenced in this section for comparison are summarized in the Supplementary Table S4. We observed that, in general, Ple628 has a lower activity with all the insoluble polyesters tested than Ple629. In different experiments, we detected the formation of BT, MHET, and T, with BT or MHET being the main degradation product. Similar studies have found that PET degradation by TfCut2, a cutinase isolated from *Thermobifida fusca* KW3, leads to the production of mainly MHET (the counterpart of BT in PET degradation) (60–70%), followed by BHET (14%) and T (8–32%) (Wei et al., 2016). Other studies also showed that some PETases, such as *Is*PETase, Mors1, PE-H and LCC, produce mainly MHET as the primary hydrolysis product rather than T after PET degradation (Yoshida et al., 2016; Bollinger et al., 2020; Blázquez-Sánchez et al., 2021; Erickson et al., 2022). However, other studies have observed T as the main hydrolysis product rather than MHET when *Is*PETase was incubated with amorphous PET films (Joo et al., 2018; Blázquez-Sánchez et al., 2021). In contrast to the assays performed by Yoshida et al. (Yoshida et al., 2016), Blazquez-Sanchez et al. (Blázquez-Sánchez et al., 2021) and Joo et al. (Joo et al., 2018), both used higher amounts of *Is*PETase (>100 nm) and longer incubation times (>24 h). This is an indication that the main source of T release was the hydrolysis of the secondary degradation from other oligomers, including MHET itself. *Is*PETase degrades MHET but at a very slow rate, and this was observed for other *Is*PETase variants as well (Brott et al., 2021).

During PET degradation, the formation of T is also possible to a lesser degree due to terminal digestions of the polymer containing T-terminal by PETase-like enzymes (Joo et al., 2018). In our study, T was produced to some extent, especially when BHET and PET-NP were supplied as the substrate. This observation suggests that BHET is a better substrate for Ple628 and Ple629 than its counterpart substrate, BHBT. During the degradation of ecovio^®^FT and its blend components, BT tended to accumulate as a degradation product. The further hydrolysis of this intermediate to T requires several hours in most cases. Similar to our study, low degradability of the hydrolysis products of PET degradation by single hydrolases has been observed before (Kleeberg et al., 2005). For instance low degradability of MHET by the cutinase HiC from *Humicola insolens* has been observed repeatedly (Carniel et al., 2017; de Castro et al., 2017). Likewise, another study performed on two cutinases, HiC and Thc-Cut1, the latter isolated from *T. cellulosilytica*, showed that HiC has poorer activity on the products released after the degradation of PBAT than Thc-Cut1, and Thc-Cut1 converted BHBT to T without the accumulation of BT (Perz et al., 2016b). In contrast to HiC, Ples have higher activity on T esterified with shorter alcohols (BHET over BHBT). Yet, HiC can degrade BHET quickly when supplied as a single substrate (Carniel et al., 2017). With PBAT, HiC cleaved butanediol-adipic acid ester bonds (B + A) better than BT (Perz et al., 2016b). It must be stressed, however, that in nature, ecovio^®^FT, PBAT, and PBSeT can be completely degraded by the action of other microbes in the consortium carrying downstream-pathway enzymes required for the complete mineralization of these biodegradable polymers (Meyer-Cifuentes et al., 2020).

The activity of Ples on ecovio^®^FT can be limited by product inhibition, e.g., BT. Similarly to our observations that BT inhibits ecovio^®^FT degradation, Barth et al. (Barth et al., 2015) found that the cutinase TfCut2 can be inhibited by the PET degradation products, MHET and BHET. Additionally, they observed that both products have a similar effect on the hydrolysis of PET by TfCut2. The same was observed for another cutinase, the LCC (Barth et al., 2016). In natural environments, the inhibition of Ples by BT can be relieved by the presence of another type of enzyme, namely Mles, as described before (Meyer-Cifuentes et al., 2020; Meyer-Cifuentes and Öztürk, 2021).

The ^inv^MM kinetics on ecovio^®^FT showed that the maximal turnover rates were achieved at 4.5 µM Ple629, after which a saturation point was reached, and the turnover rates dropped. A similar phenomenon was observed during the degradation of PET by HiC, TfC, and *Is*PETase (Bååth et al., 2021), as well as the LCC (Abhijit et al., 2018), and it is explained by the overcrowding of the enzyme surface (Mukai et al., 1993; Hiraishi et al., 2010; Bååth et al., 2021) and the rapid depletion of suitable attack sites on the enzyme surface (Scandola et al., 1998). The _inv_ *K*m value of Ple629 on ecovio^®^FT of 1.86 µM is higher than that of *Is*PETase on PET at 40°C (0.039) and TfC and HiC on PET at 50°C (0.026 and 0.043, respectively) by approximately 50-times (Bååth et al., 2021). Although the results are difficult to compare as the substrates used are different, ecovio^®^FT required more enzyme to reach saturation than PET.

Bååth et al., observed that 3-PET (denoted as BETEB in the publication) gave much higher turnover rates than amorphous PET (Bååth et al., 2021) with *Is*PETase. PBAT hydrolases Ppest (Wallace et al., 2017) and Cbotu_EstA (Perz et al., 2016a) released more products when 3-PBT (denoted as BABuTABuBA in these publications) was used than when PBAT was used. In our study, Ple629 released more products per mol enzyme when the model substrate 3-PET was used compared to nano-PET particles, but much less when 3-PBT was used compared to ecovio^®^FT and its blend components. Perz et al., also observed that the degradation rates for 3-PBT were much lower than for PBAT (Perz et al., 2016b). It has been previously demonstrated that molecules that contain more aliphatic domains are more readily degraded than those that contain a high proportion of B + T bonds, which are very rigid (Gan et al., 2004; Marten et al., 2005; Kasuya et al., 2009; Perz et al., 2016b). PBAT also has a low crystallinity (around 10%), while 3-PBT is highly crystalline, which may account for its low degradability (Perz et al., 2016b). Similar to 3-PET and 3-PBT, far more degradation products were released when BHET was used compared to BHBT, suggesting that this substrate possibly fits the enzyme active site better.

The degradation of PET-NP has been analyzed similarly with *Is*PETase and variants of *Is*PETase at different temperatures in a previous study (Brott et al., 2021). By comparing the total amount of products formed after 24 h of incubation at 30°C we observed that Ple629 presents better activity than the *Is*PETase wild type (WT) and than each variant tested in that study (Brott et al., 2021). Between 30 and 60°C, *Is*PETase WT and the different *Is*PETase variants produced less than 400 µM of MHET, T and BHET together, while Ple629 produced more than 1,200 µM of MHET and T together. The activity was comparable to the activity of an engineered high-thermal stability variant of *Is*PETase, namely DuraPETase, and its variants incubated at 60°C, which indicates that Ple629 has enhanced degradation capabilities towards PET-NP. Similarly, DuraPETase has increased degradation activity against highly crystalline PET films compared to *Is*PETase (Cui et al., 2021). Unlike *Is*PETase and DuraPETase, however, Ple629 did not degrade PET film.

The crystal structures of Ple628 and Ple629 are highly similar to those of previously characterized PETases. Both enzymes are classified as IIa PETases (Supplementary Figure S1), like the other characterized marine PETase, PE-H (Bollinger et al., 2020). Both enzymes possessed two disulfide bonds. The second disulfide bond, which is in the proximity of the active site, was shown to improve the activity of *Is*PETase (Fecker et al., 2018; Joo et al., 2018), and is also present in the other type IIa and IIb enzymes such as PE-H (Bollinger et al., 2020) and Mors1 (Blázquez-Sánchez et al., 2021) (Supplementary Figure S1).

Other activities of other tandem cutinases with high amino acid identity have previously been shown to be different, both on small substrates and plastic polymers (Herrero Acero et al., 2011; Thumarat et al., 2015; Perz et al., 2016a; Arnling Bååth et al., 2022). The tandem cutinases in these studies had even higher amino acid identity to each other than Ple628 and Ple629, demonstrating that even a few amino acid differences can lead to differences in activity in closely related enzymes. For Ple628 and Ple629, some factors that could contribute to the difference in activity are: 1) the differences in the amino acids next to the catalytic H (H252 in Ple628 and H257 in Ple629), which have been shown to influence the activity of *Is*PETase (Joo et al., 2018) and PE-H (Bollinger et al., 2020); 2) the presence of a longer extended loop in Ple629, which was previously proposed to be an extra substrate binding site for *Is*PETase (Chen et al., 2018) (Figure 6B). Likely, a combination of these factors is necessary to explain the difference in activity.

The findings of this study confirm the proposed functions of Ple628 and Ple629 as PET hydrolases with an activity on PBAT and its derivatives. These enzymes depolymerize PBAT and its derivatives, as well as PET nanoparticles to their monomers, with the aromatic terephthalate-diol monoester as the intermediate degradation product. In nature, the formed monomers and oligomers are further degraded by the action of other hydrolases, as well as oxidoreductases, lyases, etc. released by different bacteria (Sasoh et al., 2006; Yoshida et al., 2016; Li et al., 2020; Meyer-Cifuentes et al., 2020; Meyer-Cifuentes and Öztürk, 2021). In this study, Ple629 had higher catalytic activity than Ple628 both on polymeric and oligomeric substrates. In our previous study, the fold upregulation for *ple628* gene expression was higher than for *ple629* during ecovio^®^FT degradation (Meyer-Cifuentes et al., 2020). The Ple629 protein was only detected when the biofilm proteome was examined separately, and not in the whole community proteome. These findings led to the hypothesis that Ple628 played a bigger role in ecovio^®^FT degradation than Ple629. The underlying mechanism of gene expression regulation of these tandem cutinases is not known, and why the less active enzyme is produced at higher levels is unclear. Due to its much higher activity, however, we now postulate that Ple629 could also play a significant role in ecovio^®^FT degradation, although its expression levels are low, and should be included in the overall ecovio^®^FT degradation mechanism by the marine microbial consortium I1.

## 4 Materials and Methods

### 4.1 Chemicals and Substrates

Ecovio^®^FT 2341, and its blend components PBAT and PBSeT were supplied by BASF SE as plastic films. All other model substrates were in-house synthesized. The structures and synthesis protocols of the substrates BHET, BHBT, 3-PET, 3-PBT, and PET-NP are given in the Supplementary Material.

### 4.2 Recombinant Expression and Purification of Ple628 and Ple629

The signal peptides for both proteins were predicted with SignalP 5.0 (Almagro Armenteros et al., 2019). These were excluded from the final protein construct. Codon-optimized *ple628* and *ple629* were synthetically produced and cloned into the pCold II plasmid with an N-terminal His-Tag by BioCat GmbH (Germany). The plasmids were chemically transformed into *E. coli* Origami (DE3) (Merck, Germany) cells. Pre-cultures of 4-5 transformants were grown at 30°C overnight. The cultures were inoculated 1:500 into 500 ml of TB supplemented with ampicillin to an end concentration of 100 mg/L. The cultures were grown to an OD_600_ of 0.8–1 at 37°C, 140 rpm on a MaxQ™ 4,000 orbital shaker (Fischer Scientific, Germany). After induction with 0.5 mM IPTG, the cultures were grown further to an OD_600_ of 2, and then overnight at 16°C, 140 rpm. The cell pellets were collected by centrifugation at 4°C, 6,000 × g for 10 min in an Avanti J-26 XPI centrifuge with a JA-14 rotor (Beckman Coulter, United States). The pellets were frozen at −20°C for 20 min, and resuspended in His-Tag binding buffer (20 mM sodium phosphate and 0.5 M NaCl, pH 7.4) at a ratio of 1:10 of the culture volume. Cells were disrupted by sonication with a Sonopuls HD2070 sonicator (Bandelin, Germany). Cell lysates were cleared by centrifugation at 4°C 15,000 × g for 30 min in an Avanti J-26 XPI centrifuge with a JA-14 rotor (Beckman Coulter, United States) and filtrated through a 0.2 µM filter.

The proteins were purified in the same manner as described previously (Meyer-Cifuentes and Öztürk, 2021). Briefly, the cleared lysates were purified on a 5 ml His-Trap^TM^ HP affinity Ni-Sepharose column (Cytiva, Germany) connected to an ÄKTA^TM^ Start Purification System (Cytiva, Germany). Proteins were bound and eluted from the column by using the predefined affinity purification protocol included in the UNICORN^®^ start 1.1 software (Cytiva, Germany). The purity of the fractions presenting a peak was determined by SDS-PAGE. These fractions were further concentrated and desalted on a Pierce™ Protein Concentrator PES 10K MWCO (Thermo Fischer Scientific, United States). The fractions were further polished by size exclusion chromatography on a HiPrep™ 16/60 Sephacryl™ S-200 HR column (Cytiva, Germany) connected to an ÄKTA^TM^ Start Purification System (Cytiva, Germany) in a buffer consisting of 25 mM Tris–HCl and 200 mM NaCl, pH 7.5. Fractions containing the pure protein were determined by SDS-PAGE, pooled and concentrated on a Pierce™ Protein Concentrator PES 10K MWCO (Thermo Fischer Scientific, United States). The concentration of the purified protein was determined with the Qubit Protein Assay kit and measured on a Qubit 3.0 Fluorometer (Invitrogen, United States).

### 4.3 *K*m and *K*cat Determination

In a 96-well-plate, the purified Ple628 or Ple629 (end concentration 0.005 μg/μl) was combined with *p*NPA with concentrations ranging from 0.1–2.5 mM in 60 µl PBS, pH 7.0. The formation of 4-nitrophenolate was measured continuously at 405 nm at 20°C in a TECAN Infinite^®^ M200 plate reader (TECAN, Switzerland) and retrieved using the TECAN i-control v. 1.5.14.0 software (TECAN, Switzerland). The concentration of 4-nitrophenolate was calculated using a standard series in the reaction buffer and measured under the same conditions. Each assay was performed in triplicate. *K*m, *V*max and *k*cat were determined by non-linear least squares regression (http://biomodel.uah.es/en/metab/enzimas/MM-regresion.htm, last accessed 19.10.2021).

### 4.4 Activity Assay on Small Terephthalate-Esters

Activity assays with BHET and BHBT were performed in 20% DMSO in PBS, pH 7.0, to keep the substrates soluble at a final concentration of 0.5 mM. As the substrates 3-PET and 3-PBT were not soluble in this reaction buffer, they were treated as insoluble substrates, and the reactions were performed in PBS, pH 7.0. One mg of 3-PET or 3-PBT was used per assay. The final reaction volume for each experiment was 200 μl, and the final concentration of Ple628 or 629 was 0.156 µM. 50 µl of sample was taken after 1, 2, and 24 h. An additional 50 µl samples were taken for the BHET assays after 30 min. The reactions were stopped by the addition of 50 µl of cold methanol. The inactivated reactions were centrifuged for 10 min at 12,000 × g at 20°C to remove the debris, and the filtrates were transferred to HPLC vials. The degradation products BT and T were detected with a 1260 Infinity II LC System (Agilent Technologies, United States) equipped with an Agilent Poroshell 120 HPH-C18 column (Agilent Technologies, United States). The measurement protocol was modified from Palm et al., (Palm et al., 2019). A gradient of acetonitrile 99.9% HPLC grade (Fischer Scientific, United States) and 0.1% (v/v) formic acid (98–100% Suprapur^®^, Sigma-Aldrich, United States) in Milli-Q water was used. The flow rate was set at 0.2 ml/min. 10 µl of the sample was injected. Acetonitrile was increased from 5 to 44% until minute 12 and then to 70% at minute 15, remaining constant for 3 min. Both products were detected at 240 nm and the quantification was realized using calibration curves. Reactions without the enzyme were used as negative controls. Trace amounts of product formed in the negative controls were subtracted from the enzyme assay results during the calculation. Each assay was performed in triplicate.

### 4.5 Determination of Melting Points

The melting points of Ple628 and Ple629 were determined by nano differential scanning fluorimetry using the Prometheus NT.48 (NanoTemper Technologies, Germany). The measurement was conducted in ddH_2_0 and PBS buffer (pH 7.4) using a protein concentration of 1 mg/ml and a temperature profile from 20 to 95°C at 1°C/min. The instrument has a fixed excitation wavelength of 285 nm in combination with emission wavelengths of 330 and 350 nm.

### 4.6 Activity Assay on Biodegradable Plastic Films and PET-NP

To determine the optimum temperature for ecovio^®^FT degradation, 0.156 µM of Ple628 or Ple629 were incubated with 5 mg of ecovio^®^FT, supplied as film, in 500 µl of PBS at temperatures between 20–60°C, with 10°C increments. After 72 h, the concentrations of BT and T were measured as described above. Each assay was performed in triplicate.

To quantify the degradation of ecovio^®^FT, PBAT and PBSeT, 5 mg of each plastic substrate was immersed in 500 µl of PBS, pH 7.0. Ple628 or Ple629 was added to each assay tube to a final concentration of 0.156 µM. The reactions were incubated at 30 °C. 50 µl samples were taken after 24, 48, and 72 h for incubations with Ple629 and after 24 and 144 h of incubation with Ple628. The concentrations of BT and T were measured as described above. Reactions without the enzyme were used as negative controls. Each assay was performed in triplicate. Additionally, the full spectrum of degradation products was determined by LC-MS. Samples were analyzed using a 6545 Q-TOF mass spectrometer coupled to a 1290 series HPLC (Agilent, Germany) using an Acquity BEH C18 column (particle size 1.7 µM, 2.1 × 150 mm, Waters, United States). The samples were run with a 1 µl injection volume at 35°C. The eluents were A (water + 0.1% formic acid) and B (acetonitrile + 0.1% formic acid) using the following gradient: 5–44% B in 12 min, 44–70% B in 3 min, and 70% B for an additional 3 min. Compounds were detected by recording a DAD spectrum (230–640 nm) and by mass spectrometry in negative mode in a mass range of 100–1000 m/z with the following ESI settings: gas temperature 300°C, drying gas 8 L/min, nebulizer 18 psig, sheath gas 350°C and 12 L/min, capillary voltage 3 kV and fragmentor 180 V. MassHunter version B.08.00 software was used for data evaluation.

Heterogenous kinetics of ecovio^®^FT degradation by Ple629 were determined as described before using inverse Michaelis-Menten kinetics (enzyme concentration vs rate, ^inv^MM) (Scandola et al., 1998; Ronkvist et al., 2009; Wei et al., 2016; Bååth et al., 2021). To determine the ^inv^MM kinetics of Ple629, 5 mg of ecovio^®^FT film was incubated with Ple629, concentrations ranging from 0.5–6.5 µM in 500 ml of PBS, pH 7.0. 50 µl samples were taken after 6 and 24 h, and the concentrations of T and BT were measured as described above. Assays without the enzyme were used as negative controls. Each assay was performed in triplicate. The inverse *K*m (_inv_ *K*m) and *k*cat (_inv_ *k*cat) values were calculated with the linearized form of the kinetic function 1/V against 1/E0. The following formula was used:

$$\text{v}0=\frac{\text{k}cat\cdot S0\cdot E0}{\text{K}m+E0},$$ (1)

where V is the substrate degradation rate measured as (µM BT produced/h)+ (µM T produced/h), S0 is the substrate amount in mg, and E0 is the enzyme concentration in µM.

PET-NP was produced as previously described (Brott et al., 2021; Pfaff et al., 2021; Vogel et al., 2021). One hundred µg (0.2 mg/ml) PET-NP were hydrolyzed by 10 µg of Ple628 or Ple629 in a reaction volume of 500 µL. The degradation was performed in triplicates in PBS buffer (pH 7.4) at 30°C and 1,000 rpm for 24, 48, and 72 h in a ThermoMixer C (Eppendorf AG, Germany). The hydrolysis products were analyzed *via* reverse-phase HPLC on a VWR Hitachi LaChrom Elite system (VWR International, United States) equipped with a Kinetex^®^ column (5 µM EVO C18 100 Å, 150 × 4.6 mm; Phenomenex^®^, Germany). Ten µl injected sample were separated at 30°C with a gradient of acetonitrile and 0.1% (v/v) formic acid in water. Acetonitrile was increased from 5 to 44% over 12 min and then to 70% over 3 min, after which the ratio remained constant for a further 3 min, using a flow rate of 0.8 ml/min. The amounts of T, MHET, and BHET were detected *via* UV at 240 nm and the quantification was realized using calibration curves.

### 4.7 Crystallization, Data Collection, Structure Determination

The genes encoding Ple628 and Ple629 were chemically synthesized by GENE ray Biotech Co. (China) and ligated into the pET32a vector. The plasmids were transformed into *E. coli* BL21 (DE3) cells which were grown in LB medium at 37°C to an OD_600_ of 0.8 and then induced by 0.4 mM IPTG at 16°C for 24 h. Cells were harvested by centrifugation at 5,000 × g for 15 min, then re-suspended in lysis buffer containing 25 mM Tris-HCl, pH 7.5, 150 mM NaCl, and 20 mM imidazole, followed by disruption with a French Press (Thermo Electron, United States). Cell debris was removed by centrifugation at 17,000 × g for 1 h. The supernatant was then applied to a Ni-NTA column FPLC system (GE Healthcare, United States). The target proteins eluted at 100 mM imidazole when using a 20–250 mM imidazole gradient. Proteins were dialyzed against buffer containing 25 mM Tris-HCl, pH 7.5, and 150 mM NaCl, and subjected to TEV protease digestion, overnight, to remove the 6x His tag. The mixtures were then passed through another Ni-NTA column. Untagged proteins were eluted with 25 mM Tris-HCl, pH 7.5, 150 mM NaCl. After washing unbound proteins, the target protein was purified by using a DEAE Sepharose Fast Flow (Cytivia, Germany) and then a HiLoad 16/600 Superdex 200 GF column (Cytivia, Germany). The purity of proteins (>95%) was checked by SDS-PAGE analysis. The purified proteins were concentrated to 25 mg/ml for crystallization screening.

All crystallization experiments were conducted at 25°C using the sitting-drop vapor-diffusion method. In general, 1 µL Ple628 containing solution (25 mM Tris-HCl, pH 7.5, 150 mM NaCl; 25 mg/ml) was mixed with 1 µL of reservoir solution in 48-well Cryschem Plates (Hampton Research, United States), and equilibrated against 100 µL of the reservoir solution. The optimized crystallization condition of Ple628 was 20% PEG 8000, 0.2 M calcium acetate, 0.1 M sodium cacodylate, pH 6.5. The optimized crystallization condition of Ple629 (25 mM Tris-HCl, pH 7.5, 150 mM NaCl; 23 mg/ml) was 30% PEG 8000, 0.2 M ammonium acetate, 0.1 M tri-sodium citrate dihydrate, pH 5.6. Within 5–6 days, the crystals reached dimensions suitable for X-ray diffraction.

The X-ray diffraction data sets were collected at beam line BL02U1 of SSRF and BL17B, BL18U1, and BL19U1 beamline of National Facility for Protein Science in Shanghai (NFPS) at the Shanghai Synchrotron Radiation Facility (Zhang et al., 2019). The Ple628 crystals were mounted in a cryoloop and soaked with cryoprotectant solution (21% PEG 8000, 0.4 M calcium acetate, 0.1 M sodium cacodylate, pH 6.5, 20% glycerol) prior to data collection at 100 K. The cryoprotectant solution for Ple629 is 30% PEG 8000, 0.2 M ammonium acetate, 0.1 M tri-Sodium citrate dihydrate, pH 5.6, and 10% glycerol. The diffraction images were processed by using the HKL2000 software (Otwinowski and Minor, 1997). The crystal structures of Ple628 and Ple629 were both solved by the molecular replacement method with Phaser (McCoy et al., 2007) using the structure of polyester hydrolase from *Pseudomonas aestusnigri* (PDB code 6SCD) (Bollinger et al., 2020) as a search model. Further refinement was carried out using the programs Phenix v 1.19.2 (Afonine et al., 2012; Liebschner et al., 2019) and Coot (v 0.9.6) (Emsley and Cowtan, 2004). Prior to structural refinements, 5% of randomly selected reflections were set aside for calculating Rfree (Brünger et al., 1998) as a monitor. Data collection and refinement statistics are summarized in the Supplementary Table S2. All figures were prepared by using the PyMOL program (https://pymol.org/2/, last accessed on 26.10.2021). The RMSD values were calculated with Coot v 0.9.6.

### 4.8 Sequence Analysis of the PETases

The sequences used in the alignment were retrieved from the UniProt database, and aligned with the Clustal Omega 1.2.2 software built in Geneious Prime 2021.2.2.

## Data Availability

The nucleotide sequences of ple628 and ple629 were deposited in the GenBank database with the accession numbers OK558824 and OK558825, respectively.
