# Supplementary material for: Molecular and Biochemical Differences of the Tandem and Cold-Adapted PET Hydrolases Ple628 and Ple629, Isolated From a Marine Microbial Consortium
Source: Front Bioeng Biotechnol. 2022 Jul 21;10:930140. doi: 10.3389/fbioe.2022.930140 (PMC9350882; doi:10.3389/fbioe.2022.930140)
Supplement: Supplementary file 1 [file DataSheet1.pdf]

frontiers

Supplementary Material

1 SUPPLEMENTARY DATA

The synthesis of Ple substrates

MHET was synthesized according to the protocol by Palm et al. (Palm et al., 2019).

BT was synthesized according to the protocol by Perz et al. (Perz et al., 2016b), modified by Meyer-Cifuentes et al. (Meyer-Cifuentes et al., 2020). ^{1}H NMR obtained on a Bruker Avance III platform (Bruker, USA). The purity was estimated to be 93% by ^{1}H NMR (500 MHz, DMSO-d6).

3-PET was synthesized according to the protocol by Heumann et al. (Heumann et al., 2006). The purity was determined to be 95% by ^{1}H NMR (500 MHz, DMSO-d6).

3-PBT was synthesized according to the protocol by Perz et al. Perz et al. (2016b). The purity was determined to be 95% by ^{1}H NMR (500 MHz, DMSO-d6).

BHBT was synthesized using a protocol modified from Hässlin et al. (Hässlin et al., 1980). One mol 1,4-butanediol (8.84 mL) was dissolved in 20 mL of THF. A solution of 0.3 mol terephtaloyl chloride (6,1 g) and 0.45 mol pyridine (3,63 mL) in 10 mL THF was added slowly, and the reaction was brought to 40 °C. Ten more mL of THF were added to transfer the remaining solids, and the reaction was refluxed for 1h. Approximately 3/4 (30 mL) of the THF was evaporated from the reaction. The remaining solution was poured into 100 mL of ice water. The precipitate was filtered and washed with water. It was then dissolved in 30 mL of hot ethanol and filtered again to remove the undissolved residue. The solution left to cool and crystallize overnight at 4 °C. The formed crystals (the dimer of the compound) were filtered off. The filtrate was cooled down to 0 °C, filtered again and poured into ice water. The precipitated compound was filtered to obtain BHBT. The purity was determined to be 87% by ^{1}H NMR (500 MHz, DMSO-d6). 8.06 (s, 4H), 4.31 (t, 4H), 3.45 (t, 4H), 1.75 (dq, 5H), 1.59-1.50 (m, 5H).

Codon optimized sequences of Ple628 and Ple629

>Ple628
atgaatcacaaagtgcatcatcatcatcatcatatggagctcggtaccctcgagggatccgaattcgctggtggtggtggcgatggcggtga
cggtggctgcaccagtgattgtggctttcagcgtggcccggatccgaccgttagttttctggaagccagtagcggtccgtatagcgtgcgcaccg
ataatgttagtagtctggttggcggctttggcggtggcaccgttcattatccgaccggtaccaccggcaccatggcagcagtggtggttattcc
gggttttgttagtgccgaaagtagcattgaatggtggggtccgaaactggccagctatggctttgttgttatgaccattgataccaatagcggct
ttatcagccgccgagccgcgccacccagattaataatgccctggattatctgctggaagaaaatgatagcagtagtagcccgtatagtggcatga
ttgatccgaatcgcctgggtgtgattggttggagtatgggtggcggtggcacactgcgcgttgcagcagaaggccgtattcaggcagcaattccg
ctggcccgtgggataccagtagcctgcgttttcgcaatattgaaaccccgaccctgatttttgcctgcgaaagcgatgttattgcaccggtgggt
agccatgcagatccgttttatgaagccattccggatagcaccgataaagcatttttcgaactgaataatggcagccattattgcggtaatggtgg
taatagttataataatgagctgggtcgtctgggcgtgagttggatgaaactgcatctggatcaggatcagcgctataatcagtttctgtgcggtc
cggatcatgaagatgaatatcgtattagtgaataccgtggcacctgcccgtatctggaataa

>Ple629
atgaatcacaaagtgcatcatcatcatcatcatatgggtggtggtggtagtggtggtggtaataatggcggtggtggtggctgcgaagccg
attgcggttatgaacgtggcccggatccgagcgtgagcctgctggaagccagcaccggcccgtttagcgtgcgcaccagtaatgtgagtagtagc
gttcgcggctttggtggcggtaccattcattatccgaccaataccaccggtaccatggccgcaattgttgttattccgggttttgtgagcccgga
aagtagcattgcatggtggggtccgaaactggcaagtcatggctttgtggttatgaccattggtaccaatagtggctttgatcagccggccagtc
gtgcaagccagctgaataatgcactggattatctgattgaacagaatggtagtagccgcagtccgattaatggcatgattgataccgatcgtctg
ggcgttatgggctggagtatgggtggtggcggcaccctgcgtgttgcaaccgaaggtcgtgttagcgcagccattccgctggcaccgtgggatag
cagcagcagtcagtttcgtagtattgataccccgaccctgatttttgcctgtgaaaatgatagtaccgccccggttcgcagtcatgcagatccgt
tttatgatgcaattccggatagcaccgcaaaagcctttgttgaactggatggcggtggccatacctgcgccaatggtagttcaggttttggtggt
agctataatgatgttctgagccgtctgggcgtgagctggatgaaactgcatctggataaagatcagcgctataatcagtttgtttgtggtccgaa
tcatgaaagtgatcgcagtattagcgaatatcgcggtacctgcccgtatctggaataa

2 SUPPLEMENTARY TABLES AND FIGURES

2.1 Figures

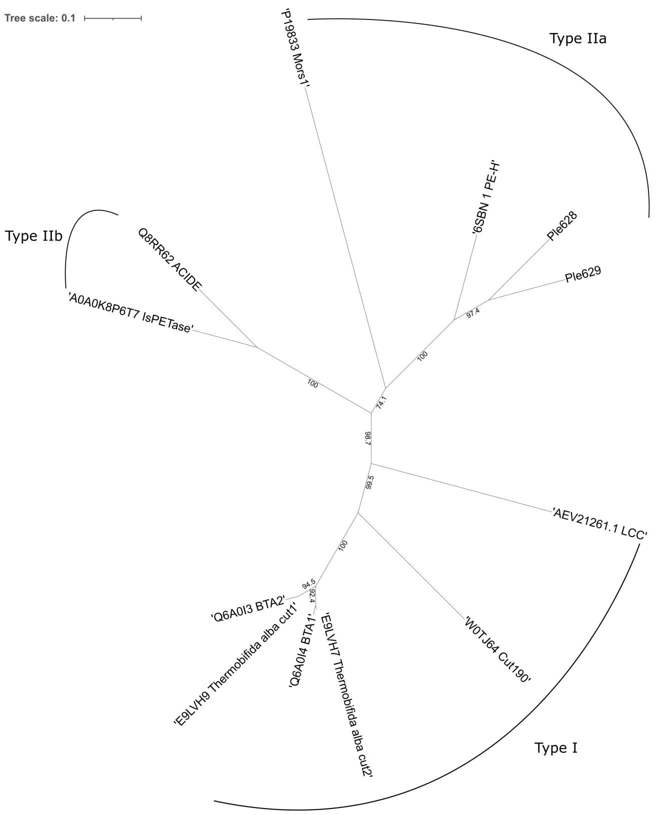

Figure S1: Phylogeny of Ple628, Ple629 and related PETases. The tree was constructed using the Geneious Prime® 2022.0.2 tree builder with the Clustal Omega protein alignment as input. The neighbour joining tree resampled 1000 times using the bootstrap method. The bootstrap values are given on the branches. The scale bar indicates amino acid substitutions per site. The UniProt IDs for each protein were indicated on the branches before the name. The PETase subtypes were assigned according to Joo et al. (Joo et al., 2018)

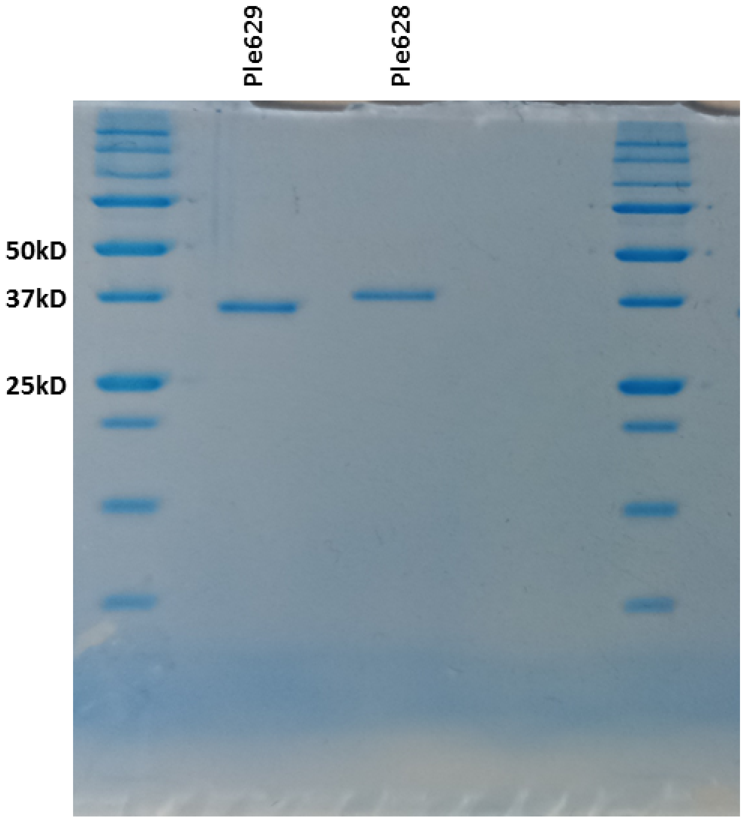

Figure S2: SDS-PAGE analysis of Ple628 and Ple629 The sizes of Ple628 and Ple629 determined experimentally by SDS-PAGE. 500 ng of protein was loaded in each well

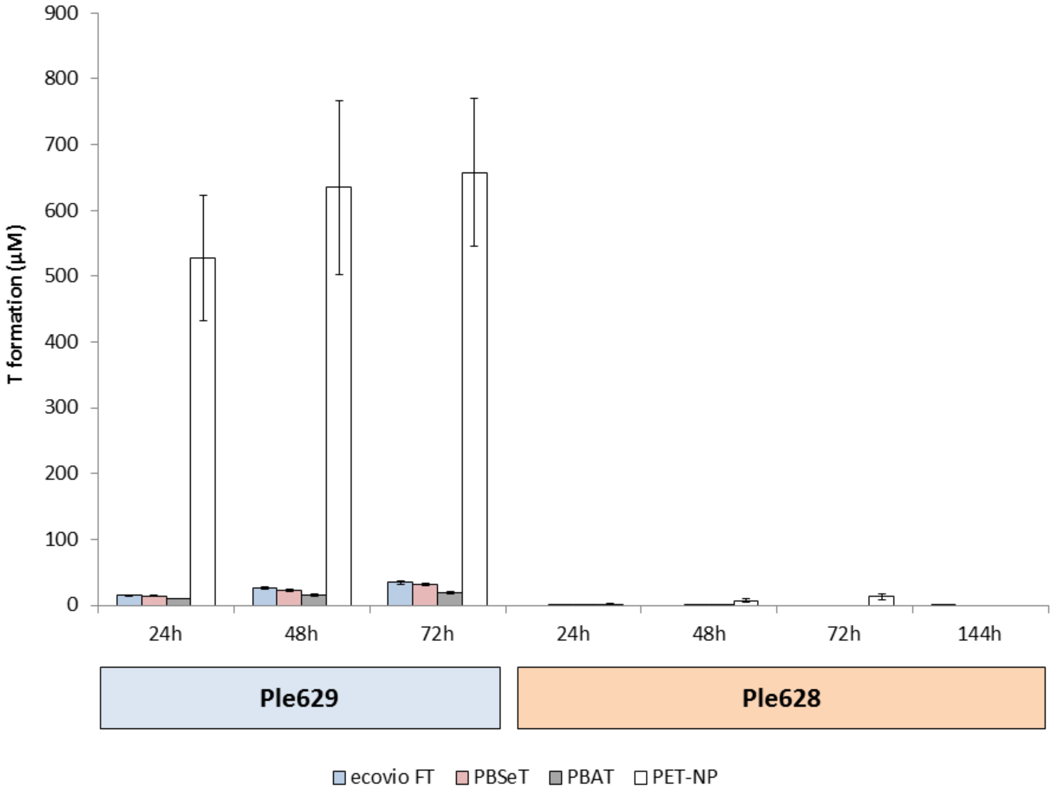

Figure S3: T formation during the degradation of PBAT-derivatives and PET. The amount of T released during the degradation of biodegradable plastics ecovio®FT, PBSeT, and PBAT as well as PET-NP, determined by HPLC. Error bars depict standard error (n = 3).

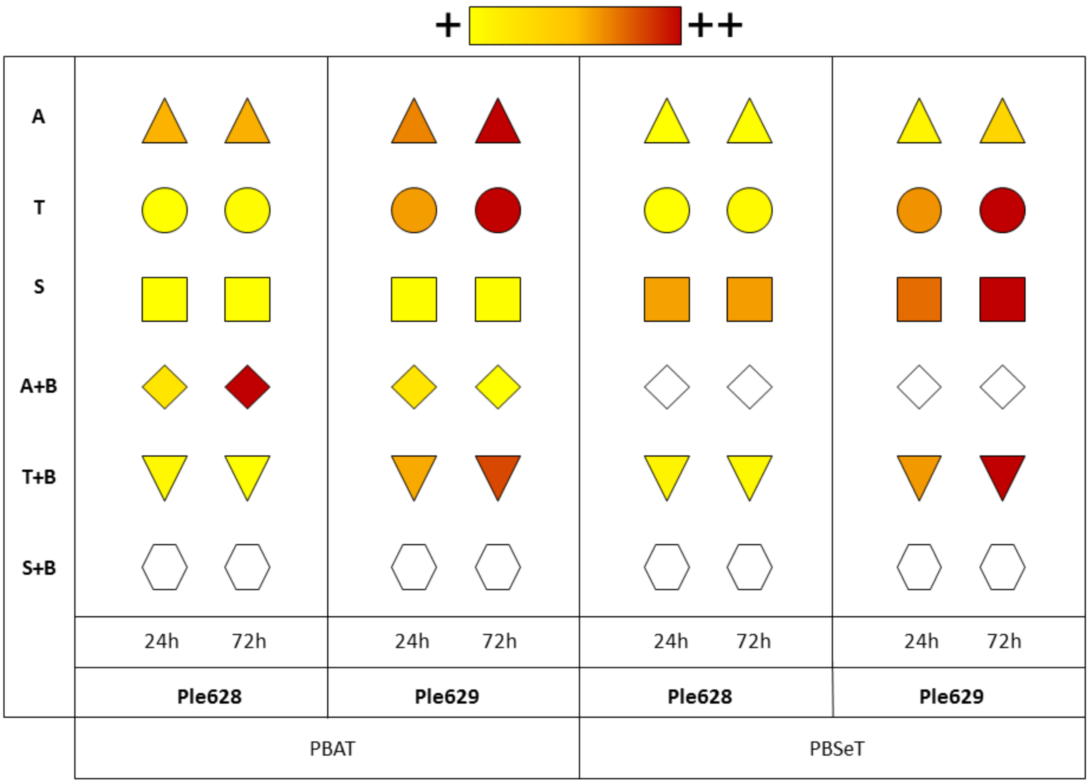

Figure S4: LC-MS-mediated relative quantification of monomer-oligomer release during plastic degradation. The gradient depicts the peak areas for each detected product, where: "A" is adipic acid; "T" is terephtalic acid; "S" is sebacic acid; "A+B" mixture of adipate and butanediol; "T + B" or BT is a mixture of terephthalic acid and butanediol; and "S + B" is a mixture of sebacic acid and butanediol . The values are averages of three replicates. Empty shapes depict undetected products.

2.2 Tables

Table S1. The Tms of Ple628 and Ple629 in different media.

Protein	Model	Variable	Value
Ple628	Two state scaled	Aw	0.177
		Tm (°C)	47.05
		ΔH (kJ/mol)	582.3
Ple629	Two state scaled	Aw	0.036
		Tm (°C)	43.16
		ΔH (kJ/mol)	774.0

Table S2. Data collection and refinement statistics of PLE crystals.

	**Ple628**	**Ple629**
Data collection		
Space group	P212121	P 32
Unit-cell		
a, b, c [Å]	43.73/64.54/81.41	73.93/73.93/90.74
α /β /γ (°)	90/90/90	90/90/120
Resolution (Å)	30.0 - 1.69 (1.75 – 1.69)	30.0 - 2.35 (2.43 - 2.35)
Unique reflections	26625 (2576)	22794 (2288)
Redundancy	8.6 (7.4)	10.5 (9.5)
Completeness (%)	99.8 (99.0)	98.6 (100)
Average I/σ(I)	12.9 (2.1)	11.9 (3.7)
CC 1/2	0.99 (0.81)	0.97 (0.73)
Refinement		
No. of reflections	26442 (2575)	22745 (2267)
Rwork (95% data)	0.159 (0.239)	0.207 (0.299)
Rfree (5% data)	0.191 (0.313)	0.262 (0.328)
Rmsd bonds (Å)	0.009	0.007
Rmsd angles (°)	1.035	0.941
Dihedral angles		
Most favored (%)	95.83	95.19
Allowed (%)	3.79	4.81
Disallowed (%)	0.38	0.00
Average B-factor/ Number of non-hydrogen atoms		
Protein	14.02/2026	32.80/4054
Solvent	25.18/363	34.99/167
Ligands	24.31/2	
PDB ID code	7VMD	7VPA

Values in parentheses are for the outermost resolution shells.

Table S3. List of referenced PET/PBAT hydrolases in the study and the substrates they have been tested on in previous studies.

Enzyme	Substrates tested	Organism	UniProt/PDB ID	Reference
HiC	BHBT, PBAT	*Humicola insolens*	A0A075B5G4	(Ronkvist et al., 2009)
Thc_Cut1	3-PET, PET	*Thermobifida cellulosilytica*	5LUI	(Herrero Acero et al., 2011)
Thf42_Cut1	3-PET, PET	*Thermobifida fusca* DSM44342	ADV92528.1	(Herrero Acero et al., 2011)
LCC	PET	Leaf compost metagenome	AEV21261.1	(Sulaiman et al., 2012)
Pe-H	PET	*Pseudomonas aestusnigri*	6SBN	(Bollinger et al., 2020)
PpEst	3-PBT, PBAT	*Pseudomonas pseudoalcaligenes*	A0A145Z9W5	(Wallace et al., 2017)
TfCut2	PET	*Thermobifida fusca* KW3	G62A	(Wei et al., 2016)
IsPETase	BHET, PET	*Ideonella sakaiensis*	5XJH	(Yoshida et al., 2016)
Cbotu_EstA	BHET, BHBT, 3-PBT, PBAT	*Clostridium botulinum*	5AH1	(Perz et al., 2016a)
Mors1	PET	*Moraxella sp.* strain TA144	P1983	(Blázquez-Sánchez et al., 2021)

REFERENCES

Blázquez-Sánchez, P., Engelberger, F., Cifuentes-Anticevic, J., Sonnendecker, C., Griñén, A., Reyes, J., et al. (2021). Antarctic polyester hydrolases degrade aliphatic and aromatic polyesters at moderate temperatures. *Appl. Environ. Microbiol.* 0, AEM.01842–21. doi:10.1128/AEM.01842-21

Bollinger, A., Thies, S., Knieps-Grünhagen, E., Gertzen, C., Kobus, S., Höppner, A., et al. (2020). A novel polyester hydrolase from the marine bacterium *Pseudomonas aestusnigri* – structural and functional insights. *Front. Microbiol.* 11, 114. doi:10.3389/fmicb.2020.00114

Hässlin, H.-W., Dröscher, M., and Wegner, G. (1980). Struktur und eigenschaften segmentierter polyetherester, 3. synthese definierter oligomerer des polybutylenterephthalats. *Makromol. Chem.* 181, 301–323

Herrero Acero, E., Ribitsch, D., Steinkellner, G., Gruber, K., Greimel, K., Eiteljoerg, I., et al. (2011). Enzymatic surface hydrolysis of PET: Effect of structural diversity on kinetic properties of cutinases from *Thermobifida*. *Macromolecules* 44, 4632–4640. doi:10.1021/ma200949p

Heumann, S., Eberl, A., Pobeheim, H., Liebminger, S., Fischer-Colbrie, G., Almansa, E., et al. (2006). New model substrates for enzymes hydrolysing polyethyleneterephthalate and polyamide fibres. *J. Biochem. Biophys. Methods* 69, 89–99. doi:10.1016/j.jbbm.2006.02.005

Joo, S., Cho, I. J., Seo, H., Son, H. F., Sagong, H.-Y., Shin, T. J., et al. (2018). Structural insight into molecular mechanism of poly(ethylene terephthalate) degradation. *Nat. Commun.* 9, 382. doi:10.1038/s41467-018-02881-1

Meyer-Cifuentes, I. E., Werner, J., Jehmlich, N., Will, S. E., Neumann-Schaal, M., and Öztürk, B. (2020). Synergistic biodegradation of aromatic-aliphatic copolyester plastic by a marine microbial consortium. *Nat. Commun.* 11, 5790. doi:10.1038/s41467-020-19583-2

Palm, G. J., Reisky, L., Böttcher, D., Müller, H., Michels, E. A. P., Walczak, M. C., et al. (2019). Structure of the plastic-degrading *Ideonella sakaiensis* MHETase bound to a substrate. *Nat. Commun.* 10, 1717. doi:10.1038/s41467-019-09326-3

Perz, V., Baumschlager, A., Bleymaier, K., Zitzenbacher, S., Hromic, A., Steinkellner, G., et al. (2016a). Hydrolysis of synthetic polyesters by *Clostridium botulinum* esterases. *Biotechnol. Bioeng.* 113, 1024–1034. doi:10.1002/bit.25874

Perz, V., Bleymaier, K., Sinkel, C., Kueper, U., Bonnekessel, M., Ribitsch, D., et al. (2016b). Substrate specificities of cutinases on aliphatic–aromatic polyesters and on their model substrates. *New Biotechnol.* 33, 295 – 304. doi:10.1016/j.nbt.2015.11.004

Ronkvist, Å. M., Xie, W., Lu, W., and Gross, R. A. (2009). Cutinase-catalyzed hydrolysis of poly(ethylene terephthalate). *Macromolecules* 42, 5128–5138. doi:10.1021/ma9005318

Sulaiman, S., Yamato, S., Kanaya, E., Kim, J. J., Koga, Y., Takano, K., et al. (2012). Isolation of a novel cutinase homolog with polyethylene terephthalate-degrading activity from leaf-branch compost by using a metagenomic approach. *Appl. Environ. Microbiol.* 78, 1556–1562. doi:10.1128/AEM.06725-11

Wallace, P. W., Haernvall, K., Ribitsch, D., Zitzenbacher, S., Schittmayer, M., Steinkellner, G., et al. (2017). PpEst is a novel PBAT degrading polyesterase identified by proteomic screening of *Pseudomonas pseudoalcaligenes*. *Appl. Microbiol. Biotechnol.* 101, 2291–2303. doi:10.1007/s00253-016-7992-8

Wei, R., Oeser, T., Schmidt, J., Meier, R., Barth, M., Then, J., et al. (2016). Engineered bacterial polyester hydrolases efficiently degrade polyethylene terephthalate due to relieved product inhibition. *Biotechnol. Bioeng.* 113, 1658–1665. doi:10.1002/bit.25941

Yoshida, S., Hiraga, K., Takehana, T., Taniguchi, I., Yamaji, H., Maeda, Y., et al. (2016). A bacterium that degrades and assimilates poly(ethylene terephthalate). *Science* 351, 1196–1199. doi:10.1126/science.aad6359
